# Editorial: Sickle cell disease in the 21st century—Morbidity to molecular biology

**DOI:** 10.3389/fmed.2022.1100846

**Published:** 2022-12-13

**Authors:** Dipika Mohanty

**Affiliations:** Apollo Hospitals Bhubaneswar, Bhubaneswar, India

**Keywords:** sickle cell disease, molecular biology, newborn screening, iron overload, pain in sickle cell disease

This Research Topic intends to review some recent developments in the management of sickle cell disease. It is now 112 years since the first description by James Herrick in 1910, while studying dentistry in Chicago, of a student from Grenada in the West Indies and knowledge of the disease has increased exponentially. The worldwide distribution of the genes contributing to sickle cell disease is now largely documented and the early assumption that the disease was confined to peoples of African descent has been rectified. Although the disease observed in the US, UK, Europe, Brazil, and the Caribbean is still predominantly among people of African descent, its presence and variability in other racial groups are increasingly recognized. These have provided models for understanding more about the disease as shown by the Asian haplotype which characterizes the disease in the Eastern Province of Saudi Arabia and throughout much of central, west and southern India. In these areas, the persistence of fetal hemoglobin (HbF) and often high frequencies of alpha thalassaemia have modified the disease which may present different features and require different models of care. The powerful contribution provided by DNA technology has also increased our understanding.

Against this background, contributions to the current topic have originated from the US, France, French Guiana, Oman, Brazil, and India. Some relate to transfusion therapy, which although sometimes beneficial in the short term, may present serious problems in chronic transfusion programmes including alloimmunization and iron overload. An IFN α/β gene signature in the majority of patients with SCD is described with the hypothesis that IFN α/β activity may determine the variable disease progression, a concept requiring further research. The use of determining EVS (Extra Cellular Vesicles) as a biomarker for sickle cell crisis is also proposed.

These topics and the mechanisms for red cell exchange are addressed and other papers look at features of chronic inflammation and chronic joint pain. Newborn screening for the disease, delayed by concerns that the high levels of HbF in the immediate post-natal period would obscure the genotype diagnosis, is now widely practiced and confers many benefits to patients if follow-up is ensured in specialist clinics. High-pressure liquid chromatography (HPLC) is the mainstay in most programmes, aspects of which are addressed in two papers, one from the US and the other from western and central India. The first raises the issue of differential diagnosis of a HbSS phenotype in which studies (parental genetics or DNA) may be required to exclude the essentially benign HbS/HPFH syndrome, which is more common than previously thought. The second raises important questions on the degree to which models of care for sickle cell disease are universal or whether they should be tailored to meet the geographical differences which are emerging. Although the basic pathophysiology of sickle cell disease may be common in most geographic areas, it is increasingly clear that interaction with genetic and environmental factors changes the manifestations of the disease. In regions of Africa where falciparum malaria is common, it is still unclear the extent to which malaria and its therapy influence disease pattern. The epidemiology of parvovirus B19 infection and the aplastic crisis is poorly documented and commonly undiagnosed in situations where reticulocyte counts are unavailable. Profound differences may occur in the disease associated with the Asian haplotype which is almost always characterized by high levels of HbF and often frequent alpha thalassaemia, both of which may be expected to inhibit intravascular sickling. In Odisha, these factors are associated with the persistence of splenomegaly and a current contribution suggests that splenic function is also preserved. This may be crucially important in protection against pneumococcal sepsis where susceptibility falls rapidly after the age of 3 years. The result is that pneumococcal septicaemia has never been described in Indian HbSS patients and with the sophistication of many Indian laboratories, it is extremely unlikely that the diagnosis is being missed. In India, the cost of a regular prophylaxis programme including conjugate and regular vaccines and penicillin has been estimated as US$220 per child but, in addition to the cost, the logistical difficulties of providing and monitoring such programmes is considerable. As diligent Indian practitioners seek to implement pneumococcal prophylaxis for a disease that current data suggest does not occur, it seems appropriate to ask whether this is the best use of these resources. Indian colleagues must make this decision and whether this is the optimal use of limited resources. Other potential differences of the Asian haplotype include a relative infrequency of priapism and leg ulceration. The bone pain crisis, usually due to avascular necrosis of bone marrow is a major problem of HbSS in all geographic areas and, in developed societies, has been increasingly managed by daycare and effective analgesia. Although painful, the great majority of events are not pathologically serious, allowing daycare and avoiding hospital admission. In Jamaica, all patients are advised to keep paracetamol/codeine mixtures at home, the combination offering much more effective pain control along with other measures such as keeping warm and well hydrated. In Jamaica, combined tablets are available without prescription and play a major role in the control of symptoms. Yet in some areas, only paracetamol is allowed for domestic use and if inadequate for pain relief, forces the patient to travel to the nearest health center or hospital for pain relief, a journey only likely to make the pain worse. The moral, of course, is that models of care must be tailored to the manifestations of local disease; such documentation has been underway in populations of African descent, when living in temperate climates, but has yet to be established in other societies.

Dipika Mohanty

Graham Serjeant

Dipty Jain

Lakshmanan Krishnamurti

## Author contributions

The author confirms being the sole contributor of this work and has approved it for publication.

